# Hydrothermal impacts on trace element and isotope ocean biogeochemistry

**DOI:** 10.1098/rsta.2016.0035

**Published:** 2016-11-28

**Authors:** C. R. German, K. A. Casciotti, J.-C. Dutay, L. E. Heimbürger, W. J. Jenkins, C. I. Measures, R. A. Mills, H. Obata, R. Schlitzer, A. Tagliabue, D. R. Turner, H. Whitby

**Affiliations:** 1 Geology and Geophysics, Woods Hole Oceanographic Institution, Woods Hole, MA 02543, USA; 2 Marine Chemistry and Geochemistry, Woods Hole Oceanographic Institution, Woods Hole, MA 02543, USA; 3 School of Earth, Energy and Environmental Sciences, Stanford University, Stanford, CA 94305, USA; 4 SCE, IPSL/CEA, UVSQ, CNRS, Université Paris-Saclay, Gif sur Yvette, France; 5 Aix Marseille Université, CNRS/INSU, Université de Toulon, IRD, Mediterranean Institute of Oceanography (MIO) UM 110, 13288 Marseille, France; 6 Department of Oceanography, University of Hawaii, 1000 Pope Road, Honolulu, HI 96822, USA; 7 Ocean and Earth Science, National Oceanography Centre Southampton, University of Southampton, Southampton SO14 3ZH, UK; 8 Atmosphere and Ocean Research Institute, University of Tokyo, 5-1-5 Kashiwanoha, Kashiwa, Chiba 277-8564, Japan; 9 Alfred Wegener Institute, Helmholtz-Center for Polar- and Marine Research, Am Alten Hafen 26, 27568 Bremerhaven, Germany; 10 Department of Earth, Ocean and Ecological Sciences, School of Environmental Sciences, University of Liverpool, Liverpool L69 3GP, UK; 11 Department of Marine Sciences, University of Gothenburg, 412 96 Gothenburg, Sweden

**Keywords:** GEOTRACES, hydrothermal activity, ocean biogeochemistry, trace elements and isotopes

## Abstract

Hydrothermal activity occurs in all ocean basins, releasing high concentrations of key trace elements and isotopes (TEIs) into the oceans. Importantly, the calculated rate of entrainment of the entire ocean volume through turbulently mixing buoyant hydrothermal plumes is so vigorous as to be comparable to that of deep-ocean thermohaline circulation. Consequently, biogeochemical processes active within deep-ocean hydrothermal plumes have long been known to have the *potential* to impact global-scale biogeochemical cycles. More recently, new results from GEOTRACES have revealed that plumes rich in dissolved Fe, an important micronutrient that is limiting to productivity in some areas, are widespread above mid-ocean ridges and extend out into the deep-ocean interior. While Fe is only one element among the full suite of TEIs of interest to GEOTRACES, these preliminary results are important because they illustrate how inputs from seafloor venting might impact the global biogeochemical budgets of many other TEIs. To determine the global impact of seafloor venting, however, requires two key questions to be addressed: (i) What processes are active close to vent sites that regulate the initial high-temperature hydrothermal fluxes for the full suite of TEIs that are dispersed through non-buoyant hydrothermal plumes? (ii) How do those processes vary, globally, in response to changing geologic settings at the seafloor and/or the geochemistry of the overlying ocean water? In this paper, we review key findings from recent work in this realm, highlight a series of key hypotheses arising from that research and propose a series of new GEOTRACES modelling, section and process studies that could be implemented, nationally and internationally, to address these issues.

This article is part of the themed issue ‘Biological and climatic impacts of ocean trace element chemistry’.

## 1. Introduction

Submarine hydrothermal circulation arises when seawater percolates downward through fractured ocean crust—for example, along the globe-encircling approximately 55 000 km volcanic/tectonic mid-ocean ridge system. As it penetrates downward, seawater is first heated and then undergoes progressive chemical modification through reactions with the host rock, reaching maximum temperatures that can exceed 400°C. At those temperatures, the fluids become buoyant and rise rapidly back to the seafloor where they are expelled into the overlying water column (see review in [1]). The most spectacular manifestation of seafloor hydrothermal circulation is without doubt the high-temperature (less than or equal to 405°C) vents that expel fluids from the seafloor along all parts of the global mid-ocean ridge-crest. In addition to being visually compelling, the fluids emitted from these vents also exhibit important enrichments and depletions in numerous trace elements and isotopes (TEIs) when compared with ambient seawater. Many of the dissolved chemicals released from the Earth's interior during venting precipitate upon mixing with the cold, overlying seawater, generating thick columns of black metal sulfide and oxide mineral-rich smoke—hence the colloquial name for these vents: ‘Black Smokers’. Despite their spectacular appearance, however, high-temperature vents may only represent a small fraction of the total hydrothermal heat flux close to ridge axes, which may be dominated by much lower-temperature diffuse flow exiting the seafloor at temperatures comparable to those first observed at the Galapagos vent sites in 1977 [2,3]. Further, while it is now known that high-temperature venting can occur in all ocean basins and along ridges of all spreading rates [4] there appear to be important differences in the styles of venting that arise in different geologic settings [5,6].

What is known from studies of ocean budgets for key conservative tracers (e.g. ^87^Sr/^86^Sr distributions) is that ocean water must be heated to high temperature, deep within the ocean lithosphere, with a residence time for circulation of the entire ocean volume along this pathway that is of the order of 10 My [2]. Because end-member vent-fluids then undergo dilution by a factor of approximately 10 000 : 1 within turbulently mixing buoyant plumes, immediately upon emission from the seabed [7], it has been estimated that the residence time of the oceans with respect to entrainment into hydrothermal plumes should be much shorter: of the order of 1000–10 000 years. This is directly comparable to the mixing time for the thermohaline conveyor and, consequently, it has long been hypothesized that processes active within deep-ocean hydrothermal plumes may influence global-scale TEI ocean biogeochemical cycles (e.g. [1,8]).

On 9 and 10 December 2015, the authors of this paper formed the membership of a break-out group focused upon *Hydrothermal Fluxes* as part of the larger *Quantifying Fluxes and Processes in Trace-Metal Cycling in the Oceans* scientific meeting hosted by the Royal Society in Chicheley Hall. After reviewing the key discoveries already made since the start of GEOTRACES (§2) the group moved on to identify some key barriers to *quantifying* the impact of hydrothermal inputs to the oceans (§3) which, in turn, allowed us to identify a series of high priority future activities, to be implemented at the scale of national and international collaborative programmes, to progress this exciting field (§4).

## 2. Hydrothermal trace element and isotope fluxes to the oceans: novel insights from GEOTRACES

**Figure 1.**
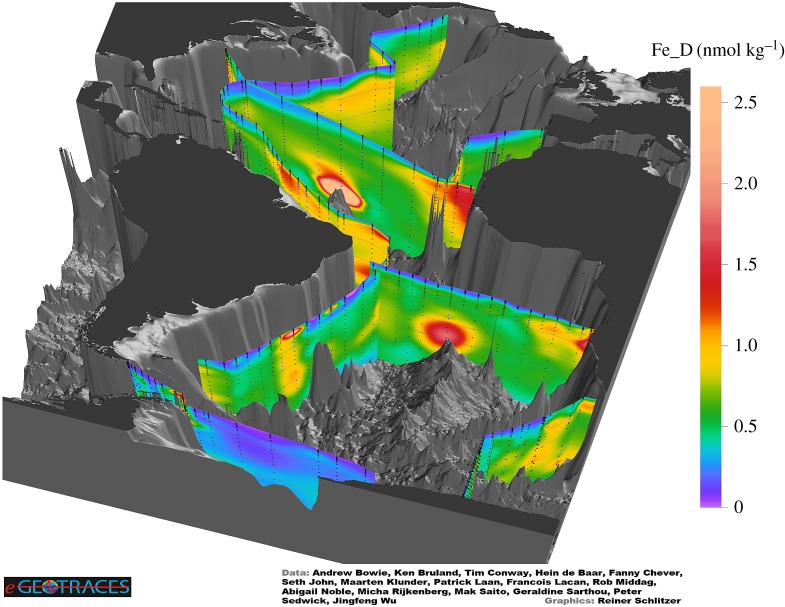
The distribution of dissolved Fe in the Atlantic Ocean based on data from the GEOTRACES Intermediate Data Product [15]. Note the presence of Fe-rich plumes centred upon, but dispersing away from the Mid-Atlantic Ridge axis.

The first discoveries of seafloor hydrothermal venting were made just as the field component of the GEOSECS programme (predecessor to GEOTRACES) was drawing to a close [9]. Consequently, scientists could only speculate (however presciently) on the importance of hydrothermal inputs to ocean biogeochemistry, at that time [10]. By contrast, the potential importance of submarine hydrothermal venting has been an important point of focus since the inception of the GEOTRACES programme [11] where mid-ocean ridges were recognized as one of four locales critical to understanding TEI fluxes to and from the oceans, alongside atmospheric deposition, continental, shelf and margin inputs and the sediment–water boundary [12–14]. In keeping with programme priorities and objectives, those ocean sections that have been occupied within the GEOTRACES programme to date [15] have included deep-ocean investigations of TEI distributions wherever ocean sections cross mid-ocean ridge crests. An important first result, arising from that work, is that a number of cruises have now provided independent and objective confirmation that hydrothermal activity is, indeed, active in all ocean basins releasing high concentrations of dissolved Fe (dFe) into the overlying water column, which can be dispersed long distances into the surrounding oceans [16–24]. Importantly, these results include clear evidence for input from hydrothermal sources along ultra-slow and slow-spreading ridges in the Arctic and Atlantic Oceans (figure 1).

Such slow-spreading ridges constitute approximately 50% of the global mid-ocean ridge axis but, historically, had been considered as unlikely to host significant venting [25]. By contrast, the new results help to confirm a more modern understanding that venting is much more abundant than previously recognized along slow-spreading ridges [4]. Further, these results take on an important socio-economic significance when one considers that such hydrothermal vent sites along slow and ultra-slow ridges may be particularly prominent, worldwide, in terms of both the size of the seafloor massive sulfide (SMS) deposits that they generate and also the high gold and copper concentrations associated with those SMS deposits [6].

**Figure 2.**
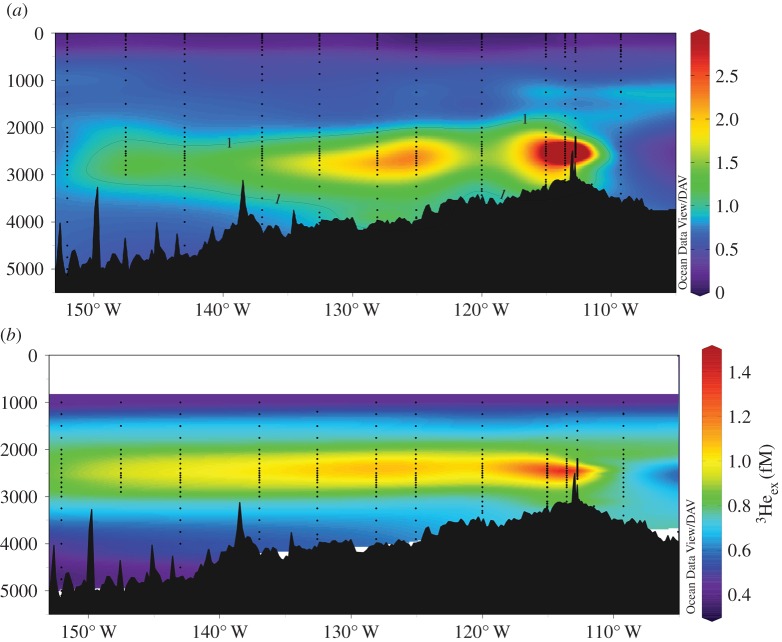
Cross-sections of (*a*) dissolved Fe and (*b*) excess dissolved ^3^He in the hydrothermal plume dispersing west from the East Pacific Rise near 15° S (data from [24]).

Most recently, the GP-16 section completed by the US GEOTRACES programme included, as one focus, an investigation of a deep hydrothermal plume spanning the south Pacific Ocean which was first identified from helium isotope analyses [26] and which, as subsequent compilations that included the GEOSECS and WOCE datasets have confirmed, represents one of the most prominent of such features worldwide [27]. Shipboard analyses from that GEOTRACES cruise have now demonstrated that dFe can be dispersed over length scales of thousands of kilometres into the ocean interior (figure 2). A particular significance of these results comes from the increasing recognition that the fate of hydrothermally sourced Fe may be intimately associated with organic carbon [28–35]. This, in turn, has stimulated particular interest in the oceanographic research community: Fe can be more than 1 million-fold enriched in vent-fluids, compared with deep-ocean waters, and yet it also acts as an essential micronutrient that limits photosynthetically driven primary productivity across up to 40% of the world's surface oceans [36]. If hydrothermally sourced Fe can persist long distances through the oceans, then its upwelling into the surface ocean could be responsible for enhancing primary productivity in the iron-limited Southern Ocean [37,38].

Of course, Fe is not the only TEI impacted by submarine hydrothermal venting. During the US GEOTRACES GA-03 section, sampling of the non-buoyant plume above the TAG hydrothermal site on the Mid-Atlantic Ridge (MAR) revealed enrichment in the plume for several elements including ^3^He [39], Fe and Mn [23], Al [40] and Hg [41]. By contrast, ^230^Th and ^231^Pa [42] showed reduced concentrations as a result of in-plume scavenging processes. This effect was also seen to reduce the concentrations of Cu [43] and Pb [44] in the plume samples compared with those immediately above or below in the water column. A small lowering of the Si isotope signal was also seen but no Si concentration anomaly was visible [45]. The fact that enriched chemical signals were also observed to the west of the MAR is consistent with the general features of material transport described by Thurnherr *et al.* [46], namely inflow from the eastern flank of the Ridge coupled with enhanced diapycnal mixing above the hydrothermal plumes, whose impact is carried westward by beta-plume dynamics [47]. Strikingly, the ^3^He : Fe ratio at TAG [39] differs substantially from that observed from the faster spreading East Pacific Rise (EPR) [24], and is more similar to that observed in the South Atlantic [19]. While the Al : Fe ratio in the TAG plume (0.73–1.15) is similar to the EPR plume (approx. 1) the Mn : Fe ratio is much lower at TAG (0.5–0.54) than in the dispersing EPR plume (1.45). At the EPR both of these values change as the plume is diluted away from the vent site (Al : Fe increases to 1.9 and Mn : Fe drops to 0.26) implying relative scavenging losses of Mn > Fe > Al in the EPR plume [24]. However, in the case of TAG it is difficult to connect these ‘far field’ signatures to specific vent sites, given the strong meridional flow in the western Atlantic, coupled with robust along rift valley flows and tidal mixing. In addition, it is likely that the off axis anomalies observed at TAG result from a significant amount of ‘integration’ from multiple other vent sites that are known to exist, north of the section [6].

The approach followed to date is useful because appropriate TEI : ^3^He ratios for hydrothermal plumes above sites of venting can be coupled with global-scale hydrothermal ^3^He fluxes to drive global biogeochemical models [48] or extrapolate and derive global TEI budgets [24,49]. That approach takes advantage of the fact that primordial ^3^He trapped in the Earth's interior leads to enrichment of ^3^He/^4^He ratios in the mantle by a factor of approximately 8 relative to atmospheric values [50,51]. These same enrichments are transferred through submarine venting to the oceans where, because He is geochemically inert in the marine environment, enrichments in ^3^He can be used as a conservative tracer of hydrothermal plume dilution and dispersion over long distances [26]. By contrast, even those TEIs that may *superficially* mimic dissolved ^3^He distributions in hydrothermal plumes (e.g. dFe, figure 2) do not act as such purely conservative tracers of physical circulation. Instead, what may be operationally defined as ‘dissolved’ Fe in a hydrothermal plume may be more complex in nature. For example, the US GEOTRACES GA-03 section crossing the TAG hydrothermal field on the northern MAR found that more than 80% of the ‘dissolved’ Fe in that dispersing plume was actually in the form of larger colloids (0.02–0.2 µm) and less than 20% was in the form of smaller colloids and soluble Fe (less than 0.02 µm) species [49]. This same topic has also been investigated through a series of more process-oriented studies, conducted close to hydrothermal vents. The results from those studies cannot currently resolve whether the colloidal Fe present is due, primarily, to the formation of organic–Fe complexes [28,30,32] or, alternatively, to the precipitation of nano-particulate Fe mineral phases [33,34]. Process studies from Southern Ocean hydrothermal plumes have demonstrated that, there, isotopic fractionation occurs during plume mixing consistent with exchange between different pools of Fe and leading to a net supply of isotopically light dFe to the oceans [52].

## 3. Hydrothermal trace element and isotope fluxes to the oceans: gaps in current understanding

To meet the goals of the GEOTRACES programme [11], we continuously seek to improve our understanding of both the inputs and impacts of hydrothermal venting on TEI ocean biogeochemistry. To identify priorities for future research, it is instructive to consider two recent studies, each of which were extrapolated from limited datasets to global-scale modelling, revealing critical gaps to our current knowledge.

First, in an attempt to model the contribution of hydrothermal Fe to the global ocean biogeochemical cycle, one approach [48] assumed a known Fe : ^3^He ratio for hydrothermal plumes which, when used to drive a global biogeochemical model, could be used to assess what proportion of Southern Ocean primary productivity might be contingent upon a hydrothermally sourced dFe supply. ^3^He is known to behave as a purely conservative tracer that can be scaled linearly with heat supply in any given hydrothermal plume, but we do not know whether all global hydrothermal systems exhibit the same ^3^He : heat supply relationship. Moreover, whether TEI : ^3^He ratios are the same in all hydrothermal systems needs to be assessed because multiple lines of reasoning suggest such might not be the case. First, a suite of early studies on medium and fast-spreading ridges indicated that axial hydrothermal heat flow might be partitioned such that only approximately 10% of that heat was released through focused high-temperature venting, at temperatures of approximately 350–400°C, while the remainder of the heat flux would be manifest in the form of dilute fluids that are emitted from the seafloor after sub-surface mixing with ambient seawater [2]. However, while the heat available from cooling of magmatic intrusions at mid-ocean ridge axes may scale with ridge spreading rate [25] what is now recognized is that slow-spreading ridges are characterized by a higher proportion of focused high-temperature fluid flow, per unit of heat flow, than their fast-spreading counterparts [53]. It is believed that this situation may arise, in part, because of differences in the partitioning of axial heat flux at slow-spreading ridges. For example, it has been estimated that the proportions of focused and diffuse flow at the MAR may be closer to 50 : 50 rather than 10 : 90 [3,54]. The case for slow- and ultra-slow-spreading ridges is complicated further by the recognition that not only do slow-spreading ridges host more high-temperature hydrothermal vents than previously anticipated [4] but, further, that at least two styles of hydrothermal venting result, each with distinct vent-fluid compositions [5,55]. An open question, therefore, is

Q1: How do the TEI : ^3^He ratios introduced into hydrothermal plumes vary as a function of the diverse styles of venting found throughout the world's oceans?

**Figure 3.**
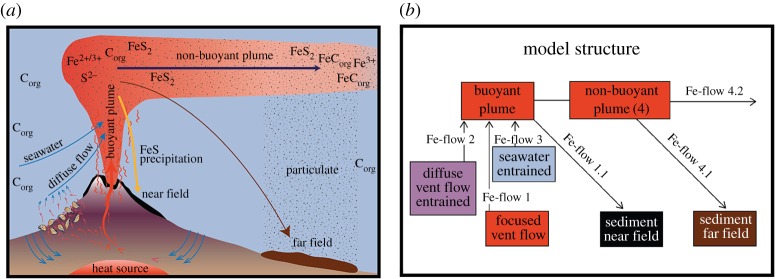
Illustration (*a*) and simplified box model (*b*) of key processes identified by SCOR-InterRidge WG 135 as critical to understanding the impact of hydrothermal venting on the deep ocean Fe and organic carbon cycle (redrawn from [35]). (Online version in colour.)

In a second recent study, SCOR Working Group 135 sought to use results from process-based investigations in near-vent environments to determine what impacts hydrothermal circulation might have on global biogeochemistry as a whole and, in particular, the global carbon budget [35]. That modelling approach revealed further important gaps in our knowledge of deep-ocean hydrothermal processes. First, while it was recognized that no one vent site on any given ridge type was likely to be representative of the global whole, insufficient data existed to populate the idealized box-model (which uses Fe as ‘currency’ to calculate organic carbon fluxes; figure 3), at any location other than the EPR 9°50′ N hydrothermal area. Further, even at that site, which had been a particular focus of international research dating from the start of the US Ridge 2000 programme, the team lacked sufficient confidence in all the required parameters to run the model in forward (prognostic) mode. Rather, because of uncertainty in the appropriate value for Fe concentrations in diffuse hydrothermal flow, the model was run in inverse mode instead. Applying global-scale Fe fluxes [24,48], this inverse approach was then used to arrive at a *predicted* representative concentration of dFe in diffuse hydrothermal fluids at the EPR. While the calculated concentrations (approx. 10–90 µmol kg^−1^) are unremarkable, a more surprising result of the same model runs was the prediction that 78–99% of all the dFe exported from hydrothermal vents to the oceans should originate from diffuse hydrothermal flow. This directly contradicts all previous expectations: because high-temperature vent fluids exhibit extremely high Fe (and other TEI) concentrations, and because some trace elements (e.g. Cu) are only mobile in solution at high temperatures in those fluids, it had been assumed that high-temperature fluid flow should dominate the TEI flux from hydrothermal venting to the oceans (e.g. [1]). The results of this modelling exercise, therefore, are quite profound. If correct, and widely representative, they may indicate an important role for organic complexation or other `pre-treatment’ processes in diffuse flow systems that allow one particular fraction of all hydrothermally sourced Fe (and other TEIs) to be protected against rapid removal into near-field sediments and, instead, exported long distances into the ocean interior. An important second question that remains open at this time, therefore, is

Q2: What is the relative importance of diffuse versus focused fluid flow in the fluxes of TEIs from hydrothermal venting to the oceans?

Finally, a third issue that arises concerns the fate of Fe and the impact that precipitation of Fe-rich particulates might have on other particle-reactive TEIs, especially given the new realization that hydrothermal venting is distributed more evenly throughout all ocean basins. While, historically, it had been assumed that hydrothermal inputs to the oceans were focused heavily toward fast-spreading ridges which are all located in the Pacific Ocean [25], we now recognize that hydrothermal inputs to the oceans are distributed more evenly in all ocean basins [4] and, hence, all along the entire thermohaline conveyor. This is important because the oxidation rate of dFe in deep-ocean hydrothermal plumes (important in driving the precipitation loss of Fe) is known to also vary systematically along the same trajectory [56,57]. While changes in the rate of oxidation and precipitation of dFe will not impact the release of a conservative tracer such as ^3^He, it may—through its impact upon dissolved–particulate Fe interactions—have an impact upon any other TEIs (e.g. rare earth elements, ^231^Pa and ^230^Th) that may be scavenged by particulate Fe phases [1]. An important third question that remains open, therefore, distinct from Question 1, which is based on varying water–rock systems, is

Q3: How do the TEI : ^3^He signatures in hydrothermal plumes vary, for individual TEIs, as a function of varying water column chemistry along the thermohaline conveyor?

## 4. Hydrothermal trace element and isotope fluxes to the oceans: a road-map toward synthesis

Moving forward, we have now acquired sufficient ocean section data to help identify a path toward synthesis and further targeted sampling that would allow the GEOTRACES programme to greatly improve our understanding of global-scale hydrothermal fluxes of TEIs to the oceans. The approach we outline for future studies continues to be so ambitious in scope that the whole could only be achieved through a level of international collaboration and coordination afforded by the international GEOTRACES programme. Below, we outline what we consider to be the highest priority activities that should be pursued, in the order: modelling, ocean sections and process studies.

### (a) Modelling

#### (i) Improving global ^3^He modelling

**Figure 4.**
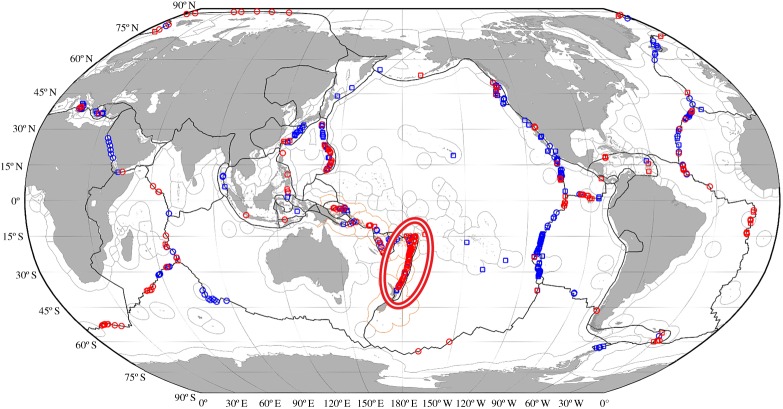
Map, modified from [58], of known hydrothermal fields along the global ridge-crest as inferred from hydrothermal plume signals (circles) or from direct seafloor observations (squares). Sites located prior to 2000 are colour-coded blue while more recent discoveries are coloured red. Note that the majority of discoveries made worldwide in the period 2000–2013 were all located in the southwestern Pacific Ocean (red and white ellipse) in a region extending from the northern Lau Basin to the North Island of New Zealand.

As discussed earlier, we have identified that combining TEI : ^3^He ratios with global fluxes of primordial ^3^He offers an extremely powerful tool for investigating the fluxes of hydrothermally sourced TEIs to the oceans and their spatial extent. While new field programmes will be required (see below) to improve our selection of the relevant TEI : ^3^He ratios, we also urge a redoubling of efforts to refine the ability of ocean physical models to reproduce the lateral dispersion patterns observed in the historic GEOSECS- and WOCE-era ^3^He datasets. Schlitzer [27] has already identified an important deficit in the modelled ^3^He input to the southwestern Pacific Ocean, suggesting that a significant source of venting is currently absent from the model in that region. Consistent with this, the latest update to the InterRidge Vents Data-Base [58] has shown that a large number of new submarine hydrothermal fields have been located throughout this portion of the global mid-ocean ridge/back-arc system with almost all of those discoveries having been made since 2000 (figure 4). Integrating those new observational data would provide a valuable first effort to further refine global dissolved ^3^He modelling. This will be important because the magnitude of the global ^3^He source is still not fully constrained. Modelling studies currently use global values that range between 450 and 1000 mol yr^−1^ [27,59–61]. However, all of those modelling studies are implemented with low horizontal and vertical resolution that, unavoidably, generates shortcomings in their simulated deep circulation. Moreover, the heat forcing from hydrothermal activity can also induce important changes in deep-ocean circulation, at both the local and global scale [47,62] and that, in turn, will affect ^3^He redistribution significantly [63]. We can improve our confidence in the value of the global ^3^He source, in future, through use of higher (vertical and horizontal) resolution models. Such models would have the capacity to reproduce the smaller scale dynamical processes discussed above which we believe to be of importance to ^3^He dispersion throughout the deep ocean.

#### (ii) Improving biogeochemical modelling

The numerous GEOTRACES ocean sections that have already intercepted deep ocean hydrothermal plumes (figure 1) [15] provide a clear indication that hydrothermal inputs of TEIs are commonplace in Earth's oceans. Where TEIs can persist in solution, upwelling along isopycnal surfaces can lead to outcropping of hydrothermally sourced TEIs in the surface ocean. Preliminary modelling investigations have calculated the impacts that hydrothermal Fe might have on Southern Ocean primary productivity [24,48]. There are also potential impacts of hydrothermally sourced TEIs on biological activity in other high-nutrient, low-chlorophyll regions of the surface ocean [27,64]. At present, however, we do not have a complete understanding of whether the TEIs injected into dispersing hydrothermal plumes are sustained in solution and disperse, like ^3^He, following isopycnal surfaces. The formation of diverse particulate phases (minerals, microbes and organic floc) that settle out of plumes and sink toward the seafloor is also an important process to consider. Ultimately, modelling of a complex series of soluble–colloidal–particulate interactions within particle-rich hydrothermal plume environments will affect the predicted scavenging of some dissolved TEIs from the oceans and their removal to the underlying sediments. By contrast, current models are only beginning to capture the full complexity of these processes [38]. A first priority for global biogeochemical models, as for the dispersion of hydrothermally sourced ^3^He (see the preceding section), would be to increase vertical resolution in the abyssal ocean to resolve the distributions of both laterally dispersing TEIs and those that settle through the water beneath dispersing hydrothermal plumes. In addition, we should also incorporate parametrizations that mechanistically resolve soluble–colloidal–particulate processes that both process studies and ocean section data are already revealing to be important [33,34,49] so that their large-scale impact can be assessed quantitatively. For example, more accurately quantifying the stability of dissolved iron organic complexes within hydrothermal plumes may be combined with emerging efforts to represent a continuum of iron binding ligands within current state of the art biogeochemical models [24,38], perhaps also accounting for pH effects [65]. Finally, while the implementation of hydrothermally sourced Fe into numerical models has greatly increased our understanding of the global Fe biogeochemical cycle [24,48], it is important to recognize that Fe is only one of the many GEOTRACES TEIs that may be influenced by seafloor hydrothermal inputs. Moving forward, a new ambition should be to assimilate the new hydrothermal data generated from GEOTRACES ocean sections into ever more sophisticated models that incorporate the biogeochemical cycles of other TEIs. Key priorities might be those TEIs that act as co-limiting micronutrients (Mn, Co and Zn), as water-mass tracers (Nd isotopes), as tracers of particle scavenging (^231^Pa/^230^Th) or nutrient cycling (N and O isotopes in nitrate) and as toxicants (Hg, Pb and As).

### (b) Ocean sections

**Figure 5.**
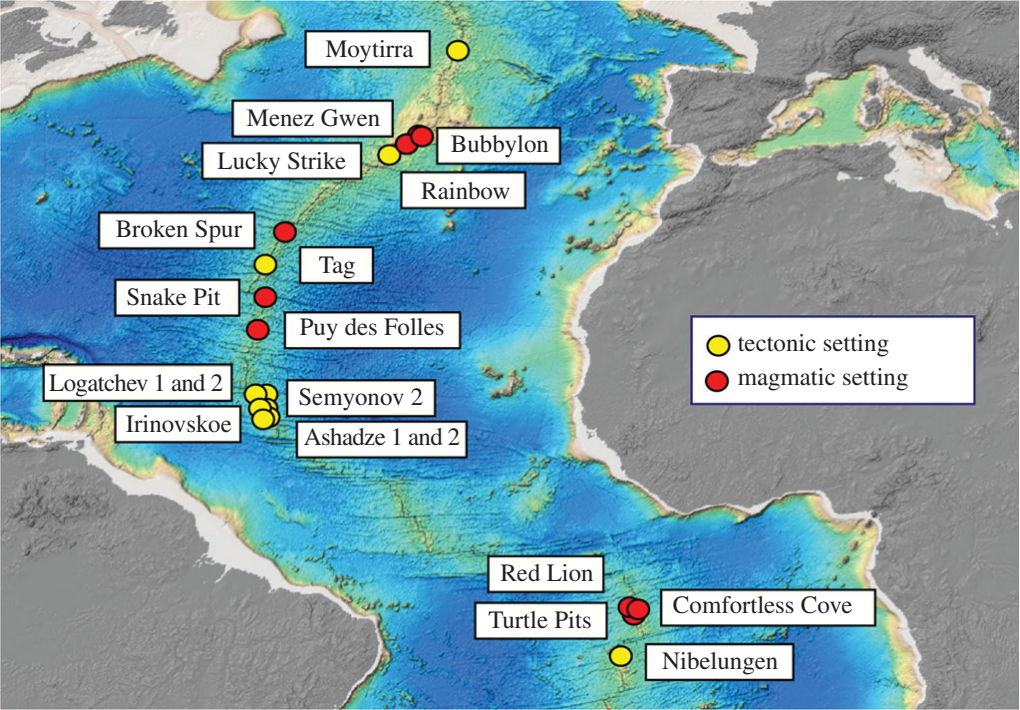
Map of all active high-temperature vent sites that have been confirmed from direct seafloor observations, to date, along the slow spreading northern Mid-Atlantic Ridge [6]. Venting extends from the Niebelungen vent site near Ascension Island, just south of the Equator to the Moytirra vent site, just north of the Azores. Colour coding differentiates between high-temperature sites of hydrothermal venting that are either in a magmatic setting (red) or a tectonic setting (yellow). Note that vent sites located in tectonic settings may be either basaltic-hosted (e.g. TAG) or ultramafic-influenced (e.g. Rainbow).

Numerous ocean sections have already been occupied within the GEOTRACES programme that cross mid-ocean ridge axes [15] and one has been targeted to follow downstream, along the dispersion trajectory of a basin-spanning hydrothermal plume [24]. A different trajectory that was never previously anticipated, but which we now recommend, is a section that follows along a portion of the mid-ocean ridge axis [11]. Slow-spreading ridges are now anticipated to host many more sites of high-temperature hydrothermal venting than was previously recognized [18,66]—so much so that it is now anticipated that there are more vent sites still to be discovered along Earth's slowest spreading ridges than have yet been discovered worldwide, despite nearly 40 years of exploration [4]. Further, the approximately 20 vent sites that have been discovered so far along the MAR (each of which emit high concentrations of TEIs into the ocean) appear to fall into two distinctive geologic categories: those set in volcanic terrain and those that are hosted, instead, under tectonic control (figure 5). This bi-modal distribution of vent-site settings is of significance to GEOTRACES because it can also give rise to important differences in hydrothermal vent-fluid compositions. On the MAR, both volcanic and tectonic-hosted systems can give rise to superficially similar ‘black smokers’ emitting fluids at temperatures of 350–400°C [5]. The presence or absence of water–rock interactions with underlying mantle rocks in these systems, however, can lead to dramatic changes in the concentrations of, for example, Fe, Cu, H_2_ and CH_4_ in the resultant vent-fluids [55]. As a first examination of how constant hydrothermal plume TEI : ^3^He ratios might be worldwide, therefore, we recommend that a dedicated ocean section be implemented, to investigate—on a like-for-like basis—how hydrothermal plume geochemistries vary in response to a range of known and geologically diverse vent sites. Specifically, a single ocean section that followed the length of the MAR, occupying stations directly above multiple known vent sites between Ascension Island and the Azores would provide immediate and valuable insights into how variable TEI : ^3^He ratios might be in hydrothermal plumes worldwide.

### (c) Process studies

Elsewhere in this issue, Jeandel *et al*. [67] use the analogy that the result of multiple geochemical or isotopic inputs to the ocean can be envisaged as the mixing of paints of varying colours within the oceans. Extending that analogy to the case of hydrothermal inputs to the oceans, our current model frameworks are rather mono-chromatic. Detailed seafloor investigations have already shown that hydrothermal circulation hosted in different geologic settings can give rise to different source vent-fluid compositions and, further, that the behaviour of dFe, one of the TEIs that is present in highest concentrations in hydrothermal vent-fluids, should be expected to behave differently—even in the case of identical source vent-fluids—depending upon where, along the global thermohaline conveyor, those vent-fluids are released into the oceans [1]. Despite our awareness of these issues, current state-of-the-art modelling of global-scale hydrothermal TEI budgets (e.g. [48]) continue to rely upon a single hydrothermal end-member. Whether this affects our large-scale understanding of the importance of hydrothermal fluxes to wider scale ocean biogeochemistry merits careful future investigation. Clearly, understanding the importance of *gross* fluxes exiting the seafloor at vent sites to broader scale ocean biogeochemistry requires detailed understanding of processes at and close to the seafloor, in addition to complementary ocean sections. A key limitation to past local-scale investigations, however, is that they have not involved a sufficiently broad range of scientists to investigate the full range of TEIs of interest to GEOTRACES. Further, even in those study areas that have received most attention, we have found that incomplete sampling has prevented us from constraining either the Fe cycle or any associated organic carbon [35].

#### (i) Selection of process study sites

Informed by knowledge gained from the Ridge 2000 programme at the EPR, 9°50′ N hydrothermal field [68] and the value it afforded to the modelling conducted by SCOR Working Group 135 [35] we now recommend that GEOTRACES should build upon that modest initial success and conduct/complete a series of detailed process-oriented studies, specifically targeting TEI cycling, in a range of ‘type locality’ geologic settings distributed along the global thermohaline conveyor. By conducting multiple studies and connecting them to process modelling, future global modelling efforts will be able to test whether varying the relative inputs from different styles of venting allows a better fit, for different TEIs, between global-scale model outputs and direct observations. This would represent an extremely valuable contribution from GEOTRACES because it would allow the relative abundances and influences of seafloor venting to be assessed, worldwide, without having to investigate the majority of the global ridge-crest. (For context, 40 years on from when the first seafloor vents were discovered, approx. 80% of the Earth's 55 000 km mid-ocean ridge systems remains to be explored, systematically, for venting.) To cover as much as possible of the known diversity of vent styles within a realistic number of target sites for an international research programme, we recommend five process study sites for investigation.

(i) A hydrothermal field located on a fast-spreading ridge and hosted in basaltic rocks is our highest priority. One candidate study site for this work would be at 15° S on the EPR where seafloor investigations could be designed to complement a recent GEOTRACES ocean section that has already completed of a full suite of TEI investigations in the overlying water column out to a range of approximately 4000 km down-plume from the source vent fields. An alternative approach might be to expand the work already conducted at the better-characterized hydrothermal vents at 9°50′ N, EPR to include a full suite of measurement types and GEOTRACES TEIs. New insights into which of these options might be preferable may come from a forthcoming GEOTRACES Pacific Meridional Transect cruise, that is expected to intercept the distal portions of both plumes (those originating at 15° S and 9°50′ N on the EPR), each of which extend at least as far as 150° W based on δ^3^He anomalies.

(ii) Our second priority is a hydrothermal field on a slow-spreading ridge that is also hosted in basaltic rocks. Our preferred candidate for this study site would be the TAG hydrothermal field at 26°08′ N on the MAR. The TAG field, like EPR 15° S, is a site that has already been visited by a GEOTRACES ocean section and has already been investigated for a full suite of TEIs in the overlying water column, both directly above and away from the vent site [69]. Further, there has already been a body of work, internationally, at and around the TAG vent site including a number of relevant biogeochemical investigations that can provide a strong basis for a more complete study of TEIs [5] using the insights provided by GEOTRACES process studies elsewhere. A further consideration is that while much of the end-member vent-fluid chemistry at TAG may be extremely similar to that found at vent sites on the EPR [70] the location of TAG on the northern MAR means that the resultant plume is injected into relatively young and oxidizing North Atlantic Deep Water. Consequently, the half-time for dFe with respect to oxidation in the TAG hydrothermal plume should be quite distinct from that found at the EPR [56]. Thus, inter-comparison of TEI cycling at TAG and an EPR vent site would also provide new insights into how hydrothermal inputs to the ocean might vary as a function of the varying chemistry of the overlying water column.

(iii) Our next priority is to investigate how changing host rock (lithology) might impact hydrothermal TEI inputs to the oceans. For slow-spreading ridges, ultra-mafic rocks represent the primary lithology other than basalts that can host hydrothermal venting, so an obvious candidate ‘type locality’ would be the Rainbow hydrothermal field at 36° N on the MAR [5]. This site, like TAG, has received much prior international attention, up to and including an early European Commission-funded attempt at quantifying export fluxes from the system [54]. Importantly, the site also allows for direct inter-comparison with the TAG hydrothermal field—both of which would ideally be included in any future along-axis MAR ocean section (see §4b, above). Because both sites are situated at comparable depths and only approximately 1000 km apart along-axis, we would predict that the Fe oxidation rates in hydrothermal plumes at both locations should be very similar. Because end-member vent-fluids also erupt at close to 360°C at both locations [70,71], therefore, we should expect that any differences in vent-fluid compositions, and in the TEI characteristics of the overlying plumes at these two sites, should be attributable primarily to changes in host rock type, and not due to changes in the overlying water column's redox poise.

(iv) To investigate the role of varying host rock still further, we also recommend that a detailed hydrothermal process study be initiated at a back-arc setting in the southwestern Pacific where abundant hydrothermal venting has been discovered in the past decade [58]. While the complex submarine geology of the southwestern Pacific can lead to a wide array of hydrothermal vent and plume types (e.g. [72]) we specifically recommend, as a candidate location for detailed process studies, the Mariner hydrothermal field at 22°10′ S on the Valu Fa ridge [73,74]. We recommend this particular site because it is here, at the southernmost limit of the Lau Basin system, where host rocks are most felsic, i.e. most distinct from the basaltic EPR. Further, the Mariner site has also already received much international attention in terms of detailed seafloor investigations, providing important underpinning for the proposed work.

(v) Finally, while the first four sites discussed above all represent differing forms of deep-water hydrothermal inputs, it is important to recognize that hydrothermal activity can also occur at much shallower depths, for example in association with island arcs and hot-spot volcanoes, and these systems may also provide significant TEI inputs to ocean biogeochemistry [75]. As with back-arc systems, island arcs exhibit a diverse geologic array that is reflected in their overlying plume chemistries (e.g. [76]). At the shallowest extreme, approximately 50 examples of marine hydrothermal vent systems have now been documented at depths of less than 200 m, releasing hydrothermal discharge directly into the photic zone [77]. While such shallow vents may have considerable impact on the biologically important coastal ocean, selecting a single ‘type locality’ that might be considered representative of such systems worldwide (hence, allowing us to make a specific recommendation for a future GEOTRACES process study) is extremely challenging. Instead, therefore, we (tentatively) suggest that an island arc site suitable for detailed process study might be the NW Rota-1 system in the Southern Mariana arc. A particular attraction of this site is that recent work has documented on-going volcanic as well as hydrothermal activity at this location [78]. Further, that work has also suggested that if NW Rota is representative of all submarine arc eruptions, this type of system may have a significant impact on the global flux of sulfur and aluminium.

#### (ii) Selection of core measurements for hydrothermal process studies

To provide realistic constraints upon the impacts of venting on TEI ocean biogeochemistry, we propose that future process studies be designed to populate the various boxes of the conceptual model originally designed for an investigation of Fe and organic carbon cycling by SCOR Working Group 135 (figure 3*b*). At each site, this would require geochemical characterization of: high-temperature vent fluids, diffuse flow fluids, buoyant hydrothermal plumes rising above the vent site, non-buoyant plumes directly above the vent site and settling particulate material sinking back toward the seafloor. In concert, at any one location, such measurements would allow the relative importance of focused versus diffuse hydrothermal flow to be assessed in terms of their role in the ocean budgets of each TEI.

By inter-comparing the results from the various ‘type localities’ outlined above, it would become possible to investigate how the relative importance of these inputs might vary as a function of different geologic settings and also in response to the evolving redox poise of the overlying water column along the thermohaline conveyor (i.e. as a function of varying [O_2_], pH and [OH^−^]).

Recognizing that hydrothermal systems have distinct characteristics, beyond those identified within the more ‘generic’ GEOTRACES science plan, we recommend that primary parameters that be selected for investigation in each process study would be: He isotopes, Fe (including both physical (e.g. size) and chemical (e.g. redox) speciation), Mn, Al, CH_4_, dissolved organic carbon (bulk and specific ligands such as siderophores), particulate organic carbon and sulfur (including S speciation). Of course, this is not to suggest that we would not want to measure the complete suite of TEI core parameters throughout the system—rather the suite of tracers here are what we consider essential to provide process-based context, capturing the anticipated diversity of hydrothermal systems that we expect might be encountered and which would be required to parametrize future, more sophisticated hydrothermal flux models. It will, for example, continue to be important to study the impact of venting upon classical tracers that have been studied elsewhere within GEOTRACES—for example, those that are representative of micronutrients (e.g. Fe), and dissolved–particulate interactions (e.g. ^230^Th/^231^Pa). Further, because hydrothermal venting may release variable quantities of key reduced species (H_2_, CH_4_ and NH_3_) that can be exploited by microorganisms [79] the relative abundances of which may vary according to lithology [80] it will also be important to track N cycling in hydrothermal vents and plumes, for example through isotopic measurements of NO_3_^−^ [81]. Finally, it will also be important to consider those tracers that may more typically be considered as ‘toxicants’ or tracers of anthropogenic pollution. Mercury is of particular interest, here, because it is illustrative of a suite of GEOTRACES TEIs for which there is still a paucity of reliable data concerning natural hydrothermal inputs to the oceans. In the specific case of Hg, hydrothermal vents are postulated to be the major *natural* source of this element to the oceans [82], but estimated contributions of hydrothermal Hg flux range over several orders of magnitude (20–2000 t yr^−1^). Consequently, quantifying the possible hydrothermal contribution to the global Hg cycle remains elusive [83–87]. To date, Hg concentrations in hydrothermal vent fluids have only been measured on three occasions [86–88] and those results show a strong site specificity, precluding direct estimations for global hydrothermal Hg fluxes. Clearly, more Hg observations of (focused and diffuse-flow) vent fluids and hydrothermal plumes are needed to better constrain the Hg flux from venting, and its contribution to the global Hg cycle. Without clear understanding of the natural inputs of such TEIs to ocean budgets, constraining the significance of anthropogenic inputs will remain a challenge.

## 5. Concluding remarks

Since the start of the GEOTRACES programme, it has become clear that hydrothermal inputs of TEIs are widespread, can occur in all ocean basins, and have the potential to be dispersed widely into the ocean interior. These findings are entirely consistent with hypotheses that had already been posed at the inception of the programme and which the design of the ocean sections phase of GEOTRACES was designed to test. Now that these fluxes have been demonstrated to exist in measurable quantities, our next priority is to quantify them and, hence, evaluate their impacts on TEIs' global biogeochemical budgets. Building upon the successes already achieved, this contribution outlines a series of recommendations for future modelling and field programmes, to achieve that goal. First, we recommend a more detailed modelling approach that incorporates the latest data for known and predicted venting, worldwide, introduces greater vertical and lateral resolution throughout the deep ocean, where hydrothermal plumes inject vent-sourced TEIs into the oceans and introduces a more sophisticated treatment of dissolved–colloidal–particulate interactions into deep-ocean modelling, in recognition that particle formation and scavenging of particle-reactive species in hydrothermal plumes may play an important role in global-scale TEI ocean biogeochemical cycles. In parallel, we propose a new body of field programmes (one section and a series of process studies) designed specifically to investigate how both the inputs and the fates of TEIs injected into the oceans from submarine hydrothermal venting might vary, both along the thermohaline conveyor (in response to varying hydrographic properties) and at ridges of different spreading rate, which provide diverse geologic settings and, hence, different vent-fluid sources.

## References

[1] German CR, Seyfried WE Jr 2014 Hydrothermal processes. In Treatise on geochemistry, 2nd edn, vol. 8 (eds Holland HD, Turekian KK), pp. 191–233. Oxford, UK: Elsevier.

[2] Elderfield H, Schultz A 1996 Mid-ocean ridge hydrothermal fluxes and the chemical composition of the ocean. Annu. Rev. Earth Planet. Sci. 24, 191–224. (10.1146/annurev.earth.24.1.191)

[3] Mottl MJ 2003 Partitioning of energy and mass fluxes between mid-ocean ridge axes and flanks at high and low temperature. In Energy and mass transfer in marine hydrothermal systems (eds Halbach P, Tunnicliffe V, Hein J). Berlin, Germany: DUP.

[4] Beaulieu SE, Baker ET, German CR 2015 Where are the undiscovered hydrothermal vents on oceanic spreading ridges? Deep-Sea Res. II Top. Stud. Oceanogr. 121, 202–212. (10.1016/j.dsr2.2015.05.001)

[5] Fouquet Y et al. 2010 Geodiversity of hydrothermal processes along the Mid-Atlantic Ridge and ultramafic hosted mineralization: a new type of oceanic Cu-Zn-Co-Au volcnogenic massive sulfide deposit. Geophys. Monogr. 188, 297–320. (10.1029/2008gm000746)

[6] German CR, Petersen S, Hannington M 2016 Hydrothermal exploration of mid-ocean ridges: where might the largest sulfide deposits be forming? Chem. Geol. 420, 114–126. (10.1016/j.chemgeo.2015.11.006)

[7] Lupton JE 1995 Hydrothermal plumes: near and far field. Geophys. Monogr. 91, 317–346. (10.1029/gm091p0317)

[8] Kadko D 1993 An assessment of the effect of chemical scavenging within submarine hydrothermal plumes on ocean geochemistry. Earth Planet. Sci. Lett. 120, 361–374. (10.1016/0012-821X(93)90250-D)

[9] Ostlund HG, Craig H, Broecker WS, Spencer D 1987 Shorebased data and graphics. GEOSECS Atlantic, Pacific and Indian Ocean expeditions, vol. 7.

[10] Edmond JM, Measures CI, McDuff RE, Chan LH, Collier R, Grant B, Gordon LI, Corliss JB 1979 Ridge crest hydrothermal activity and the balances of the major and minor elements in the ocean: the Galapagos data. Earth Planet. Sci. Lett. 46, 1–18. (10.1016/0012-821X(79)90061-X)

[11] SCOR Working Group. 2007 GEOTRACES—an international study of the global marine biogeochemical cycles of trace elements and their isotopes. Chem. Erde 67, 85–131. (10.1016/j.chemer.2007.02.001)

[12] Baker AR et al. 2016 Trace element and isotope deposition across the air–sea interface: progress and research needs. Phil. Trans. R. Soc. A 374, 20160190 (10.1098/rsta.2016.0190) 29035268 PMC5069538

[13] Charette MA et al. 2016 Coastal ocean and shelf-sea biogeochemical cycling of trace elements and isotopes: lessons learned from GEOTRACES. Phil. Trans. R. Soc. A 374, 20160076 (10.1098/rsta.2016.0076) 29035267 PMC5069537

[14] Homoky WB, Weber T, Berelson WM, Conway TM, Henderson GM, van Hulten M, Jeandel C, Severmann S, Tagliabue A 2016 Quantifying trace element and isotope fluxes at the ocean–sediment boundary: a review. Phil. Trans. R. Soc. A 374, 20160246 (10.1098/rsta.2016.0246) 29035270 PMC5069539

[15] Mawji E et al. 2015 The GEOTRACES intermediate data product. Mar. Chem. 177, 1–8. (10.1016/j.marchem.2015.04.005)

[16] Wu J, Wells ML, Rember R 2011 Dissolved iron anomaly in the deep tropical-subtropical Pacific: evidence for long-range transport of hydrothermal iron. Geochim. Cosmochim. Acta 75, 460–468. (10.1016/j.gca.2010.10.024)

[17] Klunder MB, Laan P, Middag R, de Baar HJW, van Ooijen JC 2011 Dissolved iron in the Southern Ocean (Atlantic sector). Deep-Sea Res. II Top. Stud. Oceanogr. 58, 2678–2694. (10.1016/j.dsr2.2010.10.042)

[18] Klunder MB, Laan P, Middag R, de Baar HJW, Bakker K 2012 Dissolved iron in the Arctic Ocean: important role of hydrothermal sources, shelf input and scavenging removal. J. Geophys. Res. 117, C04014 (10.1029/2011JC007135)

[19] Saito MA, Noble AE, Tagliabue A, Goepfert TJ, Lamborg CH, Jenkins WJ 2013 Slow spreading submarine ridges in the South Atlantic as a significant oceanic iron source. Nat. Geosci. 6, 775–779. (10.1038/ngeo1893)

[20] Nishioka J, Obata H, Tsumune D 2013 Evidence of an extensive spread of hydrothermal dissolved iron in the Indian Ocean. Earth Planet. Sci. Lett. 361, 26–33. (10.1016/j.epsl.2012.11.040)

[21] Conway TM, John SG 2014 Quantification of dissolved iron sources to the North Atlantic Ocean. Nature 511, 212–215. (10.1038/nature13482) 25008528

[22] Fitzsimmons JN, Boyle EA, Jenkins WJ 2014 Distal transport of dissolved hydrothermal iron in the deep South Pacific Ocean. Proc. Natl Acad. Sci. USA 111, 16 654–16 661. (10.1073/pnas.1418778111) PMC4250120 25349389

[23] Hatta M, Measures CI, Roshan S, Wu J, Fitzsimmons JN, Sedwick P, Morton P 2015 An overview of dissolved Fe and Mn distributions during the 2010–2011 U.S. GEOTRACES North Atlantic cruises: GEOTRACES GA03. Deep-Sea Res. II Top. Stud. Oceanogr. 116, 117–129. (10.1016/j.dsr2.2014.07.005)

[24] Resing JA, Sedwick PN, German CR, Jenkins WJ, Moffett JW, Sohst BM, Tagliabue A 2015 Basin-scale transport of hydrothermal dissolved metals across the South Pacific Ocean. Nature 523, 200–203. (10.1038/nature14577) 26156374

[25] Baker ET, Chen YJ, Morgan JP 1996 The relationship between near-axis hydrothermal cooling and the spreading rate of mid-ocean ridges. Earth Planet. Sci. Lett. 142, 137–145. (10.1016/0012-821X(96)00097-0)

[26] Lupton JE, Craig H 1981 A major helium-3 source at 15°S on the East Pacific Rise. Science 214, 13–18. (10.1126/science.214.4516.13) 17802550

[27] Schlitzer R 2016 Quantifying He fluxes from the mantle using multi-tracer data assimilation. Phil. Trans. R. Soc. A 374, 20150288 (10.1098/rsta.2015.0288) 29035254 PMC5069525

[28] Bennett SA, Achterberg EP, Connelly DP, Statham PJ, Fones GR, German CR 2008 The distribution and stabilization of dissolved Fe in deep-sea hydrothermal plumes. Earth Planet. Sci. Lett. 270, 157–167. (10.1016/j.epsl.2008.01.048)

[29] Toner BM, Fakra SC, Manganini SJ, Santelli CM, Marcus MA, Moffett JW, Rouxel O, German CR, Edwards KJ 2009 Preservation of iron(II) by carbon-rich matrices in a hydrothermal plume. Nat. Geosci. 2, 197–201. (10.1038/NGEO433)

[30] Sander SG, Koschinsky A 2011 Metal flux from hydrothermal vents increased by organic complexation. Nat. Geosci. 4, 145–150. (10.1038/ngeo1088)

[31] Breier JA, Toner BM, Fakra SC, Marcus MA, White SN, Thurnherr AM, German CR 2012 Sulfur, sulfides, oxides and organic matter aggregated in submarine hydrothermal plumes at 9°50′N, East Pacific Rise. Geochim. Cosmochim. Acta 88, 216–236. (10.1016/j.gca.2012.04.003)

[32] Hawkes JA, Connelly DP, Gledhill M, Achterberg EP 2013 The stabilisation and transportation of dissolved iron from high temperature vent systems. Earth Planet. Sci. Lett. 375, 280–290. (10.1016/j.epsl.2013.05.047)

[33] Yucel M, Gartman A, Chan CS, Luther GW III 2011 Hydrothermal vents as a kinetically stable source of iron-sulphide-bearing nanoparticles to the ocean. Nat. Geosci. 4, 367–371. (10.1038/ngeo1148)

[34] Gartman A, Findlay AJ, Luther GW 2014 Nanoparticulate pyrite and other nanoparticles are a widespread component of hydrothermal vent black smoker emissions. Chem. Geol. 366, 32–41. (10.1016/j.chemgeo.2013.12.013)

[35] German CR, Legendre LL, Sander SG, Niquil N, Lebris N, Luther GW III, Bharati L, Han X, Le Bris N 2015 Hydrothermal Fe cycling and deep ocean organic carbon scavenging: model-based evidence for significant POC supply to seafloor sediments. Earth Planet. Sci. Lett. 419, 143–153. (10.1016/j.epsl.2015.03.012)

[36] Boyd PW, Ellwood MJ 2010 The biogeochemical cycle of iron in the ocean. Nat. Geosci. 3, 675–682. (10.1038/ngeo964)

[37] Tagliabue A, Resing J 2016 Impact of hydrothermalism on the ocean iron cycle. Phil. Trans. R. Soc. A 374, 20150291. (10.1098/rsta.2015.0291) PMC5069527 29035256

[38] Tagliabue A, Volker C 2011 Toward accounting for dissolved iron speciation in global ocean models. Biogeosciences 8, 3025–3039. (10.5194/bg-8-3025-2011)

[39] Jenkins WJ, Lott DE III, Longworth BE, Curtice JM, Cahill KL 2015 The distributions of helium isotopes and tritium along the US GEOTRACES North Atlantic sections (GEOTRACES GA03). Deep-Sea Res. II Top. Stud. Oceanogr. 116, 21–28. (10.1016/j.dsr2.2014.11.017)

[40] Measures C, Hatta M, Fitzsimmons J, Morton P 2015 Dissolved Al in the zonal N Atlantic section of the US GEOTRACES 2010/2011 cruises and the importance of hydrothermal inputs. Deep-Sea Res. II Top. Stud. Oceanogr. 116, 176–186. (10.1016/j.dsr2.2014.07.006)

[41] Bowman KL, Hammerschmidt CR, Lamborg CH, Swarr G 2015 Mercury in the North Atlantic Ocean: the US GEOTRACES zonal and meridional sections. Deep-Sea Res. II Top. Stud. Oceanogr. 116, 251–261. (10.1016/j.dsr2.2014.07.004)

[42] Hayes CT, Anderson RF, Fleisher MQ, Huang K-F, Robinson LF, Lu Y, Cheng H, Edwards RL, Moran SB 2015 ^230^Th and ^231^Pa on GEOTRACES GA03, the U.S. GEOTRACES North Atlantic transect, and implications for modern and paleoceanographic chemical fluxes. Deep-Sea Res. II Top. Stud. Oceanogr. 116, 29–41. (10.1016/j.dsr2.2014.07.007)

[43] Jacquot JE, Moffett JW 2015 Copper distribution and speciation across the International GEOTRACES Section GA03. Deep-Sea Res. II Top. Stud. Oceanogr. 116, 187–207. (10.1016/j.dsr2.2014.11.013)

[44] Noble A, Echegoyen-Sanz Y, Boyle EA, Ohnemous CC, Lam PJ, Kayser R, Reuer M, Wu J, Smethie W 2015 Dynamic variability of dissolved Pb and Pb isotope composition from the U.S. North Atlantic GEOTRACES transect. Deep Sea Res. II Top. Stud. Oceanogr. 116, 208–225. (10.1016/j.dsr2.2014.11.011)

[45] Brzezinski MA, Jones JL 2015 Coupling of the distribution of silicon isotopes to the meridional overturning circulation of the North Atlantic Ocean. Deep-Sea Res. II Top. Stud. Oceanogr. 116, 79–88. (10.1016/j.dsr2.2014.11.015)

[46] Thurnherr AM, Richards KJ, German CR, Lane-Sherff GF, Speer KG 2002 Flow and mixing in the rift valley of the Mid-Atlantic Ridge. J. Phys. Oceanogr. 32, 1763–1778. (10.1175/1520-0485(2002)032%3C1763:FAMITR%3E2.0.CO;2)

[47] Stommel H 1982 Is the south Pacific helium-3 plume dynamically active? Earth Planet. Sci. Lett. 61, 63–67. (10.1016/0012-821X(82)90038-3)

[48] Tagliabue A et al. 2010 Hydrothermal contribution to the oceanic dissolved iron inventory. Nat. Geosci. 3, 252–256. (10.1038/ngeo818)

[49] Fitzsimmons JN, Carrasco GG, Wu J, Roshan S, Hatta M, Measures CI, Conway TM, John SG, Boyle EA 2015 Partitioning of dissolved iron and iron isotopes into soluble and colloidal phases along the GA03 GEOTRACES North Atlantic Transect. Deep-Sea Res. II Top. Stud. Oceanogr. 116, 130–151. (10.1016/j.dsr2.2014.11.014)

[50] Kurz MD, Jenkins WJ, Schilling JG, Hart SR 1982 Helium isotopic variations in the mantle beneath the central North Atlantic Ocean. Earth Planet. Sci. Lett. 58, 1–14. (10.1016/0012-821X(82)90099-1)

[51] Lupton JE, Craig H 1975 Excess ^3^He in oceanic basalts: evidence for terrestrial primordial helium. Earth Planet. Sci. Lett. 26, 133–139. (10.1016/0012-821X(75)90080-1)

[52] Lough AJM, Klar JK, Homoky WB, Milton JA, Connelly DP, James RH, Mills RA Submitted Iron isotope fractionation during hydrothermal plume mixing and dispersal in the deep ocean.

[53] Baker ET, Edmonds HN, Michael PJ, Bach W, Dick HJB, Snow JE, Walker SL, Banerjee NR, Langmuir CH 2004 Hydrothermal venting in magma deserts: the ultraslow-spreading Gakkel and Southwest Indian Ridges. Geochem. Geophys. Geosyst. 5, Q08002 (10.1029/2004GC000712)

[54] German CR, Thurnherr AM, Radford-Knoery J, Charlou J-L, Jean-Baptiste P, Edmonds HN 2010 Export fluxes from submarine venting to the ocean: a synthesis of results from the Rainbow hydrothermal field, 36°N MAR*.* Deep-Sea Res. I Oceanogr. Res. Pap. 57, 518–527. (10.1016/j.dsr.2009.12.011)

[55] Charlou JL, Donval JP, Konn C, Ondréas H, Fouquet Y, Jean-Baptiste P, Fourré E 2010 High production and fluxes of H_2_ and CH_4_ and evidence of abiotic hydrocarbon synthesis by serpentinization in ultramafic-hosted hydrothermal systems on the Mid-Atlantic Ridge. Geophys. Monogr. 188, 265–296. (10.1029/2008gm000752)

[56] Field MP, Sherrell RM 2000 Dissolved and particulate Fe in a hydrothermal plume at 9°45′N, East Pacific Rise: slow Fe(II) oxidation kinetics in Pacific plumes. Geochim. Cosmochim. Acta 64, 619–628. (10.1016/S0016-7037(99)00333-6)

[57] Statham PJ, German CR, Connelly DP 2005 Iron (II) distribution and oxidation kinetics in hydrothermal plumes at the Kairei and Edmond vent sites, Indian Ocean. Earth Planet. Sci. Lett. 263, 588–596. (10.1016/j.epsl.2005.03.008)

[58] Beaulieu SE, Baker ET, German CR, Maffei A 2013 An authoritative global database for active submarine hydrothermal vent fields. Geochem. Geophys. Geosyst. 14, 4892–4905. (10.1002/2013GC004998)

[59] Farley KA, Maier-Reimer E, Schlosser P, Broecker WS 1995 Constraints on mantle ^3^He fluxes and deep-sea circulation from an oceanic general circulation model. J. Geophys. Res. 100, 3829–3839. (10.1029/94JB02913)

[60] Dutay J-C et al. 2004 Evaluation of OCMIP-2 ocean models’ deep circulation with mantle helium-3. J. Mar. Syst. 48, 15–36. (10.1016/j.jmarsys.2003.05.010)

[61] Bianchi D, Sarmiento JL, Gnanadesikan A, Key RM, Schlosser P, Newton R 2010 Low helium flux from the mantle inferred from simulations of oceanic helium isotope data. Earth Planet. Sci. Lett. 297, 379–386. (10.1016/j.epsl.2010.06.037)

[62] Adcroft A, Scott J, Marotzke J 2001 Impact of geothermal heating on the global ocean circulation. Geophys. Res. Lett. 28, 1735–1738. (10.1029/2000GL012182)

[63] Dutay J-C, Emile-Geay J, Iudicone D, Jean-Baptiste P, Madec G, Carouge C 2010 Helium isotopic constraints on simulated ocean circulations: implications for abyssal theories. Environ. Fluid Mech. 10, 257–273. (10.1007/s10652-009-9159-y)

[64] Tagliabue A, Williams RG, Rogan N, Achterberg EP, Boyd PW 2014 A ventilation-based framework to explain the regeneration-scavenging balance of iron in the ocean. Geophys. Res. Lett. 41, 7227–7236. (10.1002/2014GL061066)

[65] Avendaño L, Gledhill M, Achterberg EP, Rérolle VMC, Schlosser C 2016 Influence of ocean acidification on the organic complexation of iron and copper in Northwest European Shelf Seas; a combined observational and model study. Front. Mar. Sci. 3, 58 (10.3389/fmars.2016.00058)

[66] Edmonds HN, Michael PJ, Baker ET, Connelly DP, Snow JE, Langmuir CH, Dick HJB, German CR, Graham DW 2003 Discovery of abundant hydrothermal venting on the ultraslow-spreading Gakkel Ridge in the Arctic Ocean. Nature 421, 252–256. (10.1038/nature01351) 12529639

[67] Jeandel C 2016 Overview of the mechanisms that could explain the ‘Boundary Exchange’ at the land–ocean contact. Phil. Trans. R. Soc. A 374, 20150287. (10.1098/rsta.2015.0287) PMC5069524 29035253

[68] Fornari DJ et al. 2012 The East Pacific Rise between 9°N and 10°N: twenty-five years of integrated, multidisciplinary oceanic spreading center studies. Oceanography 25, 18–43. (10.5670/oceanog.2012.02)

[69] Boyle EA, Anderson RF, Cutter GA, Fine RA, Saito M, Jenkins WJ 2015 Introduction to the U.S. GEOTRACES North Atlantic Transect (GA-03): USGT10 and USGT11 cruises. Deep Sea Res. II Top. Stud. Oceanogr. 116, 1–5. (10.1016/j.dsr2.2015.02.031)

[70] Edmond JM, Campbell AC, Palmer MR, Klinkhammer GP, German CR, Edmonds HN, Elderfield H, Thompson G, Rona P 1995 Time-series studies of vent-fluids from the TAG and MARK sites (1986, 1990) Mid-Atlantic Ridge: a new solution chemistry model and a mechanism for Cu/Zn zonation in massive sulphide ore-bodies. Geol. Soc. Spec. Publ. 87, 77–86. (10.1144/GSL.SP.1995.087.01.07)

[71] Charlou JL, Donval JP, Fouquet Y, Jean-Baptiste P, Holm N 2002 Geochemistry of high H_2_ and CH_4_ vent fluids issuing from ultramafic rocks at the Rainbow hydrothermal field (36°14′N, MAR). Chem. Geol. 191, 345–359. (10.1016/S0009-2541(02)00134-1)

[72] Lupton J, Rubin KH, Arculus R, Lilley M, Butterfield D, Resing J, Baker E, Embley R 2015 Helium isotope, C/^3^He, and Ba-Nb-Ti signatures in the northern Lau Basin: distinguishing arc, back-arc and hotspot affinities. Geochem. Geophys. Geosyst. 16, 1133–1155. (10.1002/2014GC005625)

[73] Takai K et al. 2008 Variability in the microbial communities and hydrothermal fluid chemistry and the newly discovered Mariner hydrothermal field, southern Lau Basin. J. Geophys. Res. 113, G02031 (10.1029/2007JG000636)

[74] Mottl MJ et al. 2011 Chemistry of hot springs along the Eastern Lau Spreading Center. Geochim. Cosmochim. Acta 75, 1013–1038. (10.1016/j.gca.2010.12.008)

[75] Hawkes JA, Connelly DP, Rijkenberg MA, Achterberg EP 2014 The importance of shallow hydrothermal island arc systems in ocean biogeochemistry. Geophys. Res. Lett. 41, 942–947. (10.1002/2013GL058817)

[76] Resing JA, Baker ET, Lupton JE, Walker SL, Butterfield DA, Massoth GJ, Nakamura K 2009 Chemistry of hydrothermal plumes above submarine volcanoes of the Mariana Arc. Geochem. Geophys. Geosyst. 10, Q02009 (10.1029/2008GC002141)

[77] Prol-Ledesma RM, Dando PR, de Ronde CEJ 2005 Special issue on ‘shallow water hydrothermal venting’. Chem. Geol. 224, 1–4. (10.1016/j.chemgeo.2005.07.012)

[78] Butterfield DA, Nakamura K, Takano N, Lilley MD, Lupton JE, Resing JA, Roe KK 2011 High SO_2_ flux, sulfur accumulation, and gas fractionation at an erupting submarine volcano. Geology 39, 803–806. (10.1130/G31901.1)

[79] Dick GJ, Anantharaman K, Baker BJ, Li M, Reed DC, Sheik CS 2013 The microbiology of deep-sea hydrothermal vent plumes: ecological and biogeographic linkages to seafloor and water column habitats. Front. Microbiol. 4, 124–128. (10.3389/fmicb.2013.00124) 23720658 PMC3659317

[80] Reveillaud J, Reddington E, McDermott J, Algar C, Meyer JL, Sylva S, Seewald J, German CR, Huber JA 2016 Subseafloor microbial communities in hydrogen-rich vent fluids from hydrothermal systems along the Mid-Cayman Rise. Environ. Microbiol. 18, 1970–1987. (10.1111/1462-2920.13173) 26663423 PMC5021209

[81] Bourbonnais A, Lehmann MF, Butterfield DA, Juniper SK 2012 Subseafloor nitrogen transformations in diffuse hydrothermal vent fluids of the Juan de Fuca Ridge evidenced by the isotopic composition of nitrate and ammonium. Geochem. Geophys. Geosyst. 13, Q02T01 (10.1029/2011GC003863)

[82] Sonke JE, Heimburger LE, Dommergue A 2013 Mercury biogeochemistry: paradigm shifts, outstanding issues and research needs. C. R. Geosci. 345, 213–224. (10.1016/j.crte.2013.05.002)

[83] Fitzgerald WF, Engstrom DR, Mason RP, Nater EA 1998 The case for atmospheric mercury contamination in remote areas. Environ. Sci. Technol. 32, 1–7. (10.1021/es970284w)

[84] Mason RP, Sheu GR 2002 Role of the ocean in the global mercury cycle. Glob. Biogeochem. Cycles 16, 1093 (10.1029/2001GB001440)

[85] Fitzgerald WF, Lamborg CH 2004 Geochemistry of mercury in the environment. In Treatise on geochemistry, vol. 9 (eds Holland HD, Turekian KK), pp. 1–47. Oxford, UK: Elsevier.

[86] Lamborg CH, Von Damm KL, Fitzgerald WF, Hammerschmidt CR, Zierenberg R 2006 Mercury and monomethylmercury in fluids from Sea Cliff submarine hydrothermal field, Gorda Ridge. Geophys. Res. Lett. 33, L17606 (10.1029/2006GL026321)

[87] Crespo-Medina M, Chatzeiefthimiou AD, Bloom NS, Luther GW, Wright DD, Reinfelder JR, Vetriani C, Barkay T 2009 Adaptation of chemosynthetic microorganisms to elevated mercury concentrations in deep-sea vents. Limnol. Oceanogr. 54, 41–49. (10.4319/lo.2009.54.1.0041)

[88] Sherman LS, Blum JD, Nordstrom DK, McCleskey RB, Barkay T, Vetriani C 2009 Mercury isotopic composition of hydrothermal systems in the Yellowstone Plateau volcanic field and Guaymas Basin sea-floor rift. Earth Planet. Sci. Lett. 279, 86–96. (10.1016/j.epsl.2008.12.032)

